# Exploring the relationship across autonomous motivation, affects, and anxiety among gym practitioners during the second COVID-19 lockdown

**DOI:** 10.1038/s41598-024-57878-2

**Published:** 2024-03-27

**Authors:** Raúl Antunes, Filipe Rodrigues, Miguel Jacinto, Nuno Amaro, Rui Matos, Diogo Monteiro

**Affiliations:** 1 ESECS - Polytechnic University of Leiria, Leiria, Portugal; 2 Research Center in Sport Sciences, Health Sciences and Human Development (CIDESD), Vila Real, Portugal; 3 Life Quality Research Centre, Leiria, Portugal

**Keywords:** Motivation, COVID-19, Fitness, Mental health, Physiology, Psychology

## Abstract

The present study explores the association of autonomous motivation and the relationship of positive and negative affect on anxiety levels among individuals engaged in gym practitioners during the second COVID-19 lockdown. A total of 196 exercisers (29.17 ± 10.77) were enrolled in the present study, of which 112 (57.1%) were women and 84 (42.9%) were men. The survey included sociodemographic data, as well as validated instruments measuring autonomous motivation, positive and negative affect, and anxiety states related to the COVID-19 pandemic. The results revealed a positive association between autonomous motivation and positive affect (β = 0.36, CI 0.12, 0.37; *p* < 0.001), and a negative association between autonomous motivation and negative affect (β = − 0.17, CI − 0.31, − 0.01; *p* = 0.03). Moreover positive, and negative affect are negatively (β = − 0.33, CI − 0.43, − 0.24; *p* < 0.001) and positively (β = 0.72, CI 0.57, 0.82; *p* < 0.001) associated to anxiety, respectively. Thus, this study appears to emphasize the association of autonomous motivation on affect as a potential buffer against anxiety levels, particularly in a context where practitioners found themselves restricted in their usual gym practices.

## Introduction

The COVID-19 pandemic, caused by the SARS-CoV-2 virus, started in December 2019 in the city of Wuhan, China. The World Health Organization declared the new coronavirus a public health emergency^1^. The Portuguese government declared a state of emergency on 18 March and, consequently, a set of preventive public health measures were gradually implemented^2^. The lockdown in Portugal took place between March and June 2020 (Decree no. 2-A/2020, of 20 March, Resolution of the Council of Ministers no. 40-A/2020, of 29 May), and then from January to April 2021 (Decree no. 3-A/2021, of 14 January, Resolution of the Council of Ministers no. 19/2021, of 13 March). During these periods, gyms and sports clubs were closed in Portugal. While these measures were considered vital to prevent the spread of the virus and can have a positive effect on protecting people's physical health, they can also have consequences for mental health, for example higher levels of anxiety^3,4^. In the same sense, social confinement can lead to an increase in sedentary behavior and anxiety levels^5^ and the frustration of basic psychological needs^6^. Knowing that regular physical exercise can be an effective tool to promote mental health, including reducing anxiety levels and increasing well-being^7^, especially in the context of COVID-19^5,8^, it is essential to understand the role of behavioral regulation during the period of lockdown in the practice of exercise by individuals.

### Motivation towards exercise

Motivation can play a crucial role in the maintenance of exercise during the COVID-19 lockdown. During this challenging period, motivation can stem from various sources. Some people may find motivation in the desire to maintain good physical and mental health, especially during a pandemic^9^. The Self-Determination Theory (SDT), initially proposed by Deci and Ryan in 1985^10^ and further elaborated in 2017^11^, can be a valuable framework for understanding motivation during the COVID-19 lockdown, especially in the context of exercise and health. At its core, SDT explores the concept of motivation and discerns three distinct forms: amotivation, controlled motivation and autonomous motivation^12^. At its core, SDT explores the concept of motivation and discerns three distinct forms: amotivation, controlled motivation, and autonomous motivation. Amotivation is characterized by a lack of motivation, where individuals exhibit disinterest or a sense of detachment from engaging in a particular behavior. Controlled motivation involves external or internal pressures that drive behavior, such as the need for rewards or avoiding punishment. Autonomous motivation, on the other hand, represents a self-driven, intrinsic desire to engage in a behavior. This form of motivation arises from personal choice, interest, or inherent satisfaction derived from the activity itself. Autonomous motivation is particularly crucial as it has been linked to sustained effort, higher levels of well-being, and increased persistence in the face of challenges^13,14^. Understanding the implications of autonomous motivation is key in promoting long-term adherence to behaviors like exercise and well-being, where individuals are more likely to sustain their efforts when driven by a genuine internal desire rather than external or internal pressures.

### Correlates of motivation

Autonomous motivation is particularly crucial due to its strong association with well-being, influencing both positive and negative dimensions^15,16^. Subjective well-being, a multidimensional construct linked to factors influencing personal equilibrium and health, has been recognized as a positive dimension of health. According to Diener^17^, it involves the presence of positive aspects, the absence of negatives, and a positive perception of life satisfaction. This concept includes affective and cognitive dimensions, where affective dimension relates to experienced positive and negative affects^18,19^. Positive affect refers to a state in which an individual experience feeling of excitement, activity, and alertness. It is associated with a sense of high energy, heightened concentration, and overall pleasure. On the other hand, negative affect is regarded as a subjective dimension characterized by distress and the absence of pleasurable feelings. This dimension encompasses various aversive states of mind, including feelings of guilt, fear, nervousness, contempt, and anger. There is some empirical evidence supporting a positive correlation between autonomous motivation and positive affect in the realm of exercise. When individuals autonomously choose to partake in physical activity, they often experience elevated levels of enjoyment, satisfaction, and excitement, contributing to an overall positive affect^16^. Moreover, the impact on negative affect is noteworthy. Autonomous motivation tends to mitigate the presence of negative emotions, such as stress or anxiety, as individuals feel a sense of choice and volition in their decision to engage in exercise. Thus, there would be a negative association between autonomous motivation and negative affect^20^.

Existing literature posits that positive affect exhibits a notable buffering effect against anxiety, a phenomenon attributed to its adaptive influence on cognitive and emotional processing^21^. Empirical evidence suggests that individuals experiencing heightened positive affect may demonstrate enhanced coping mechanisms and greater resilience when confronted with stressors^22^. This resilience, in turn, is postulated to mitigate the propensity for anxiety development. Thus, the psychological resources conferred by sustained positive affect may contribute to the attenuation of anxiety. Conversely, negative affect, comprising emotions such as fear, sadness, and anger, has been consistently associated with an increased susceptibility to anxiety disorders^20^. The proclivity for rumination and excessive worry, integral components of anxiety pathology, is notably exacerbated in the presence of sustained negative affect^23^. In light of the unprecedented global circumstances engendered by events such as the COVID-19 pandemic, the relevance of investigating the intricate interrelationships between affective states and anxiety becomes particularly salient.

The lockdown has led to heightened stressors, increased uncertainty, and disruptions to individuals' daily lives, thereby amplifying the prevalence of anxiety^24^. Understanding the predictors of anxiety during such a challenging period becomes imperative for informing targeted interventions and support mechanisms. In this vein, investigating the role of psychological factors, such as autonomous motivation, positive affect, and negative affect, holds relevance. Existing literature highlights that positive affect plays a crucial role in mitigating anxiety, especially in uncertain circumstances such as the alleviation of lockdowns^25,26^. On the other hand, negative affect has been negatively associated with anxiety, affecting mental well-being^27–29^.

### Current study

The current investigation seeks to shed light on the intricate relationships between autonomous motivation, anxiety, and affective dimensions during a period of lockdown. Changes in daily life routines during confinement can lead to an increase in sedentary and inactive behavior, anxiety levels and negative affect^30^. These sedentary and inactive lifestyles increase anxiety symptoms and impair mental health and well-being^7^. Autonomous forms of motivation and physical activity, with an identified tendency towards regulation, show a stronger association with initial or short-term adoption than intrinsic motivation. However, intrinsic motivation emerges as a better predictor of long-term adherence to exercise. Overall, the literature reports evidence that supports the importance of SDT in understanding exercise behavior, showing the crucial role of autonomous regulation in encouraging physical activity^31^. Knowing these relationships, namely that practicing physical activity reduces anxiety and improves well-being and that autonomous motivation acts as a predictor of adherence to physical exercise, assessing the influence of autonomous motivation on anxiety is pivotal for unraveling the protective effects associated with self-determined behaviors. In particular, the study aims to discern the nuanced associations between autonomous motivation and both positive and negative affect, and subsequently, their impact on anxiety. The examination of the link between autonomous motivation and anxiety holds significant relevance, considering the potential buffering role of autonomous motivation behaviors in mitigating anxiety, especially in the context of restrictive conditions such as a lockdown. This investigation aligns with the broader discourse on the psychological dynamics influencing mental health outcomes during periods of confinement^16,32^. Furthermore, delving into the associations between autonomous motivation and positive affect, as well as autonomous motivation and negative affect, forms a crucial aspect of this study. Preliminary evidence suggests a positive association between autonomous motivation and positive affect, and conversely, a negative association with negative affect. Unraveling these relationships contributes to a nuanced understanding of the emotional landscape associated with self-determined behaviors, offering insights into the affective correlates of autonomous motivation^33,34^. The examination of positive and negative affect as predictors of anxiety is an additional facet of interest. Positive affect is hypothesized to act as a protective factor, buffering against anxiety, while negative affect is anticipated to exacerbate its presence. This exploration aligns with established theories on the role of affective states in shaping mental health outcomes and is particularly pertinent in the unique context of exercise during confinement. Moreover, the study endeavors to address a notable gap in the literature by investigating the association between these variables in the specific context of exercisers during a period of confinement. The intersection of autonomous motivation, affective states, and anxiety in the exercise domain within the confines of a lockdown is a novel avenue for exploration. Lastly, the investigation aims to contribute to the understanding of potential mediating mechanisms. Specifically, it seeks to elucidate the mediating roles of positive and negative affect in the association between autonomous motivation and anxiety. Despite emerging clues in the literature, this aspect remains unclear, and the study endeavors to provide empirical insights into the intricate pathways through which affective states may mediate the impact of autonomous motivation on anxiety^16,35^.

A comprehensive examination of autonomous motivation, positive affect, and negative affect as predictors of anxiety during the COVID-19 lockdown is essential for advancing our understanding of the psychological factors at play and informing targeted mental health interventions. Thus, the present study explores the influence of autonomous motivation and the relationship of positive and negative affect on anxiety levels among exercisers during the second COVID-19 lockdown.

## Method

### Participants

The requisite sample size was calculated employing the Daniel Sopper online calculator^36^, incorporating the following input parameters: anticipated effect size (0.3), statistical power level (0.80), number of latent variables (2), and number of observed variables (9). Consequently, the determined minimum sample size for effect detection was 119, the minimum sample size for the model structure was 89, and the recommended minimum sample size of 119 was adhered to in the present study. Accounting for a margin of 10% missing data and/or outliers, we incorporated an additional 10% to the minimum sample size, resulting in a total minimum sample size of 98 participants being collected.

In the current study, a cohort of 196 exercisers (M = 29.17; SD = 10.77) was included, comprising 112 females (57.1%) and 84 males (42.9%). During to the lockdown, participants reported a weekly training frequency of 3.81 (SD = 1.60) sessions. The inclusion criteria encompassed individuals aged 18 years and above, willing to participate voluntarily, and possessing an active membership of at least 6 months. Participants were explicitly made aware of their option to withdraw from the study at any time. Furthermore, participants were required to provide their consent before the conclusion of the survey, with the research commitment to ensuring anonymity throughout the process.

### Measures

The Behavioral Regulation in Exercise Questionnaire Portuguese version^37^ designed to evaluate various motivational regulations served as an instrument in this study. Specifically, our investigation focused on dimensions of autonomous motivation, namely identified regulation (“I value the benefits of exercise”), integrated regulation (“I exercise because it is consistent with my life goals”), and intrinsic motivation (“I exercise because it's fun”). Respondents rated these dimensions on a 5-point Likert scale, ranging from 0 (“totally disagree”) to 4 (“totally agree”), in response to the stem: “Why do you engage in exercise?” Their responses reflected how they perceived and actualized their motivation during exercise. Previous studies, such as the work conducted by Rodrigues et al.^38^, validate the effectiveness of the scale in assessing behavioral regulations within the Portuguese exercisers. We computed the autonomous motivation factor by averaging the latent means, following the approach proposed by Howard^39^. In the current study, the internal consistency of the subscales exhibited strong reliability, with alpha coefficients of α = 0.81 for identified regulation, α = 0.93 for integrated regulation, and α = 0.93 for intrinsic motivation.

To assess the experience of positive and negative affect, we applied the Positive and Negative Affect Schedule Portuguese version^40^. This version comprises ten items, introduced by the stem “To what extent do you feel each of the emotions when training,” prompting participants to indicate their responses on a five-point Likert scale, ranging from 1 (“totally disagree”) to 5 (“totally agree”). The 10-item version is recommended for studies focusing on positive (e.g., “inspired”) and negative (e.g., “nervous”) affect measures^16^. The internal consistency for the subscales in this study demonstrated good reliability, with alpha coefficients of α = 0.83 for positive affect and α = 0.85 for negative affect.

The State-Trait Anxiety Inventory Portuguese version^41^ was employed as the tool to evaluate state anxiety. Specifically, only the 20 items measuring anxiety state dimension were used, with scores ranging from 20 (minimum) to 80 (maximum) and has shown acceptable validity in the exercise population^5^. The anxiety state dimension assesses temporary or transient anxiety, reflecting the participant's current emotional state (e.g., “I’m worried”). The internal consistency of this factor for this study demonstrated good reliability alpha coefficient of α = 0.62.

### Procedure

The collection of data adhered to the principles outlined in the Helsinki Declaration^42^, with approval granted by the Ethics Committee of the Polytechnic of Leiria (CE/IPLEIRIA/35/2021) for its implementation. The data collection procedures took place in February and March 2021, coinciding with the second lockdown in Portugal. Recruitment of potential participants and study promotion were carried out through social media. An online questionnaire was constructed and made available to potential participants. Specifically, we targeted gym practitioners for participation. The study objectives were thoroughly explained to participants. Prior to the assessment of the questionnaire, informed consent was obtained from all subjects.

### Data analysis

Initially, means and standard deviations were computed, along with skewness and kurtosis, and bivariate correlations across all variables in the study. Hair^43^ argued that data is considered to be normal if skewness is between − 2 to + 2 and kurtosis is between − 7 to + 7. These analyses were conducted using IBM SPSS STATISTICS version 29 (IBM Corp).

Following this, a two-step procedure according to Kline^44^ was executed using IBM SPSS AMOS version 27. In the first step, a measurement model was estimated through confirmatory factor analysis evaluate the fit of the measurement model to the data. Next, the structural model was developed to test the study hypotheses. The full information robust maximum likelihood estimator was employed to address the limited missing data at the item level (random missing = 3%), following the approach recommended by Enders^45^. Subsequently, we proceeded with the analysis of descriptive statistics and bivariate correlations. The adequacy of both the measurement and structural models was evaluated employing traditional incremental indices, namely Comparative Fit Index (CFI) and Tucker-Lewis Index (TLI), along with absolute indices, specifically Standardized Root Mean Residual (SRMR) and Root Mean Square Error of Approximation (RMSEA) with its corresponding confidence interval. Cutoff values recommended by various authors^43,46^ were adopted: CFI and TLI ≥ 0.90; RMSEA and SRMR ≤ 0.08. The chi-square (χ2) split degrees of freedom (df) are reported for transparency, but not examined, since both are affected by model complexity and sample size^43^.

During the assessment of psychometric properties, we also conducted a convergent validity analysis, determined by the average extracted variance (AVE) with coefficients ≥ 0.50^43,47^, assuming values at or above this threshold as acceptable. Discriminant validity analysis was deemed adequate when the square of the correlations between factors was lower than the AVE of each factor^43^, and these values were specifically calculated. Internal consistency for each latent variable was evaluated using composite reliability, considering coefficients at or above 0.70 as adequate^48^.

As a result of the structural equation model, standardized direct and indirect effects on the dependent variable were examined. The significance of these effects was determined using a bootstrap resampling procedure (1000 bootstrap samples) with a 95% confidence interval (CI). An indirect effect was considered significant (*p* ≤ 0.05) if the 95% CI did not include zero^49^.

## Results

The descriptive results in Table 1 indicate that participants exhibited above the midpoint levels of autonomous motivation and positive affect. Additionally, participants reported moderate levels of anxiety. Scores of skewness and kurtosis were within limits of − 2/ + 2 and − 7/ + 7, respectively suggesting normal distribution of all factors. Bivariate correlations revealed a significant pattern across all the variables under study. The most pronounced bivariate correlation was noted between negative affect and anxiety (r = 0.70, *p* < 0.01), while the weakest correlation was observed between autonomous motivation and negative affect (r = − 0.21, *p* < 0.01).

**Table 1:** Descriptive statistics and bivariate correlations.

Variables	M	SD	S	K	1	2	3	4
1. Autonomous motivation	3.25	0.87	1.02	2.96	1	–	–	–
2. Positive affect	3.42	1.98	0.34	0.82	0.42*	1	–	–
3. Negative affect	1.96	0.84	0.89	1.34	− 0.21*	− 0.26*	1	–
4. Anxiety state	42.76	11.02	1.72	3.12	− 0.25*	− 0.58*	0.70*	1

M, mean; SD, standard deviation; S, Skewness; K, Kurtosis.

*p < 0.01.

The measurement model test incorporates three latent and one observed variable interrelated with each other: autonomous motivation, positive and negative affect, and anxiety, respectively. The results of the measurement model demonstrated a good fit to the data: [χ^2^/*df* = 2.68, B-S *p* < 0.001, TLI = 0.922, CFI = 0.941, SRMR = 0.052, RMSEA = 0.066 (CI 0.058, 0.070)]. Regarding psychometric properties, the results indicated satisfactory convergent validity (AVE > 0.50), as well as appropriate values for discriminant validity, where the square of the correlations between the factors was lower than the value of variance extracted from each factor and adjusted internal consistency values exceeded 0.70 (Table 2). Therefore, these findings support the initial conditions necessary for conducting the structural model and analyzing the direct and indirect effects among the studied variables.

**Table 2:** Convergent and discriminant validity, and internal consistency coefficients.

Variables	AVE	1	2	3	4	CR
1. Autonomous motivation	0.85	1	–	–	–	0.94
2. Positive affect	0.53	0.14	1	–	–	0.83
3. Negative affect	0.54	0.03	0.04	1	–	0.85
4. Anxiety state	–	0.004	0.19	0.42	1	–

AVE, Average variance extracted; below the diagonal line = squared latent correlations; CR, composite reliability coefficients.

**p < 0.01.

**Figure 1:**
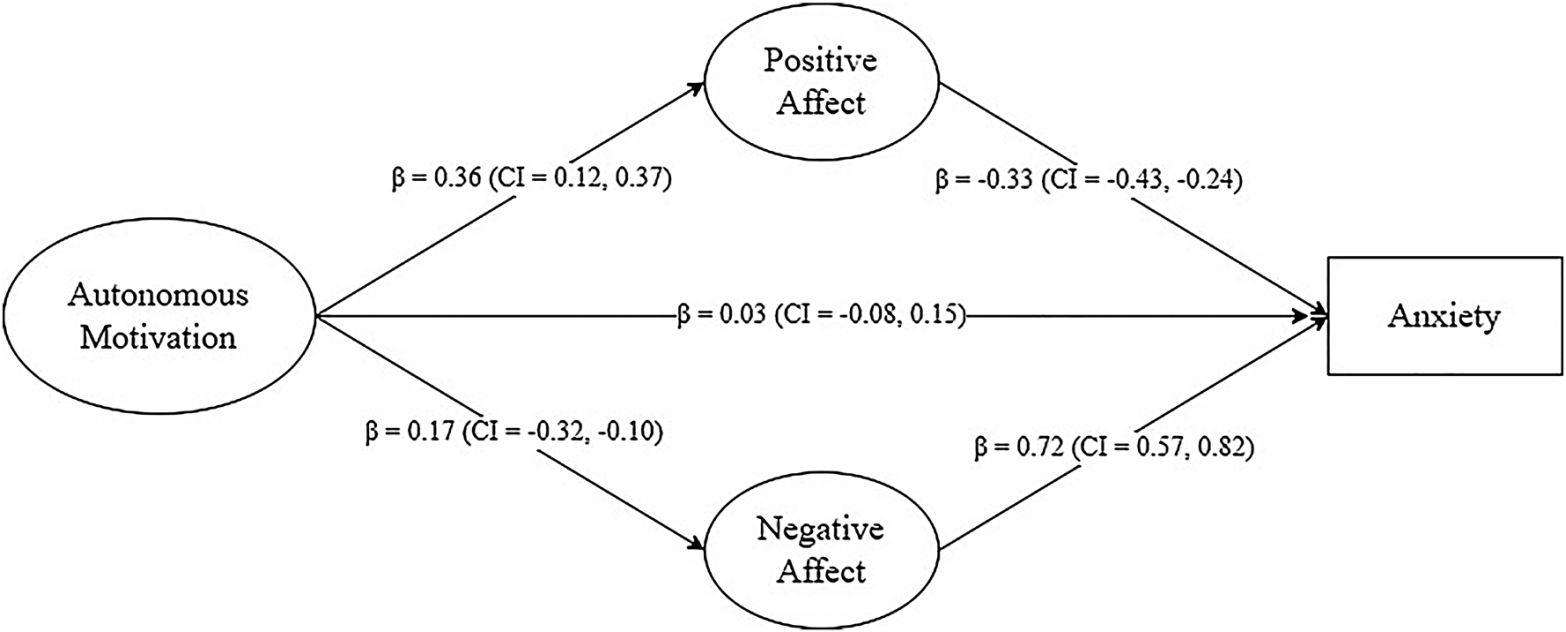
SEM Model. *Notes*: β = standardized coefficient; CI = Confidence Interval; *p* = level of significance.

The results of the structural model indicated an acceptable fit to the data: [χ^2^/*df* = 2.78, B-S *p* < 0.001, TLI = 0.908, CFI = 0.937 SRMR = 0.054, RMSEA = 0.069 (CI 0.060, 0.073)]. Autonomous motivation exhibits both negative and positive associations with negative affect and positive affect, respectively. Moreover, positive, and negative affect are negatively and positively linked to anxiety. The standardized direct (β = 0.03, CI − 0.08, 0.15; *p* = 0.52) and indirect effects between autonomous motivation and anxiety through negative affect (β = − 0.02, CI − 0.16, 0.12; *p* = 0.78) and positive affect (β = − 0.08, CI − 0.04, 0.032; *p* = 0.62) are negatively and positively, respectively but not significant. In total, the model explained variance on anxiety was 57%. For details see Fig. 1.

## Discussion

The present study explored the influence of autonomous motivation and the relationship of positive and negative affect on anxiety levels among gym practitioners engaged in exercises during the second COVID-19 lockdown.

Descriptive statistics results show lower values of anxiety compared to Taiwan adults^50^, in confinement situations. Similarly, in a representative sample of college students, the results of our participants for anxiety was also lower^51^. On the other hand, physical activity has a direct effect on anxiety, in another sample of studies of physical activity participants^52^. Regarding affects, the participants of our study show higher values of both affects comparatively the participants with youth aged^53^. In a student’s populations, descriptive statistics show lower values of positive affect and higher values of negative affect when capered with our results. The results are similar for an adult sample (including participants who practiced physical activity). At the same time, the participants who were physically active during the lockdown period reported a higher level of positive affect. On the other hand, in a study of exercisers, the values for positive and negative affect were similar to those we found, with frequency of exercise showing a positive and significant association with positive affect^35^. The same can be seen when analyzing elderly people who practice physical activity^54^.

Physical activity/exercise seems reduces anxiety and improves well-being. However, lockdown has increased sedentary and inactive lifestyles, increased anxiety and decreasing well-being in the general population^30^. Knowing that a more autonomous motivation promotes engagement in and maintenance of behaviors such as exercise, which in turn impacts anxiety and well-being, it is important to study these relationships in exercisers^55^.

Our results indicated that autonomous motivation exhibits a significant association with affective states, manifesting both negative and positive associations with negative and positive affect, respectively. The observed associations are aligned with established theories, as outlined in the existing literature^5,11^. Our empirical findings affirm the theoretical assertions regarding the relationship between autonomous motivation and affect. The positive correlation identified between autonomous motivation and positive affect aligns with existing evidence in the exercise domain. Engaging in physical activity driven by autonomous motivation is associated with elevated levels of enjoyment, satisfaction, and excitement, contributing to an overall positive affect^13,16,31^. Moreover, the observed negative association between autonomous motivation and negative affect is in line with theoretical expectations^16^. Autonomous motivation appears to act as a mitigating factor against negative emotions, such as stress, as individuals exercising volitional choice in their physical activity engagements experience a diminished presence of distress^5,16^.

The associations revealed in our study between positive affect, negative affect, and anxiety align with the theoretical underpinnings outlined in the introduction and extend our understanding of psychological factors influencing mental health during the COVID-19 lockdown^33^. As mentioned in the introduction, the unprecedented global situation during the lockdown has significantly heightened stressors and uncertainties, leading to an increased prevalence of anxiety. Positive affect, characterized by heightened feelings of excitement, activity, and pleasure, exhibits a negative association with anxiety, confirming existing literature that underscores the anxiety-mitigating role of positive affect during uncertain circumstances, such as the lockdown^5,56^. Individuals experiencing higher positive affect may find themselves better equipped to cope with stressors and uncertainties, thereby reducing the likelihood and severity of anxiety. Conversely, our study reveals a positive association between negative affect and anxiety. This aligns with the established literature highlighting the adverse impact of negative affect on mental well-being, particularly its role in exacerbating anxiety^57,58^. The heightened prevalence of negative emotions, including distress and aversive states of mind, corresponds to an increased likelihood of experiencing anxiety during challenging periods like the COVID-19 lockdown. Thus, the affective states experienced during exercise shape emotional responses that extend beyond the immediate context, with positive affect influencing the overall emotional landscape and contributing to lower levels of anxiety^59^. Furthermore, the positive affect developed through regular exercise contributes to psychological resilience, enabling more effective coping with stressors and anxiety-inducing situations in various life domains^60^. These psychological mechanisms collectively influence the broader experience of anxiety at a global psychological level.

The examination of the mediating role of positive and negative affect in the relationship between autonomous motivation and anxiety reveals nuanced findings. Despite a negative bivariate correlation between autonomous motivation and anxiety state (see Table 1), our study indicates that the standardized indirect effects between autonomous motivation and anxiety through both negative and positive affect are negative but not statistically significant (Fig. 1). The presence of positive and negative affect does not significantly mediate the relationship between autonomous motivation and anxiety. The non-significant indirect effects underscore the complexities of the relationship between autonomous motivation and anxiety during a challenging period like the lockdown. While the negative bivariate correlation aligns with expectations^61,62^, the lack of a significant mediation effect by positive and negative affect seems suggests that the impact of autonomous motivation on anxiety may operate independently of these affective states. On the other hand, although the relationships between the variables are significant but the indirect effects are not, this could be related to variables or relationships not considered in the final model or to the insufficient size of the sample.

### Limitations and agenda for future research

The current study, while providing valuable insights into the associations among autonomous motivation, affective states, and anxiety during the unique circumstances of the COVID-19 lockdown, is not without its limitations. Firstly, the study adopts a cross-sectional design, restricting our ability to establish causality or temporal relationships among the variables under investigation. Secondly, the absence of a comparison with pre-lockdown and post-lockdown periods limits our capacity to discern the specific impact of the lockdown itself on the observed relationships. A comparative analysis would have provided valuable insights into whether the identified associations are specific to the lockdown conditions or are more enduring. The dynamics of outdoor exercise, influenced by environmental factors and differing regulations, may have yield distinct results. Additionally, our study exclusively explored the influence of a single negative emotion, namely anxiety. While anxiety is a pertinent mental health indicator, future research should expand its scope to incorporate a more comprehensive array of positive emotional states.

### Practical implications

The imposed limitation on regular exercise during lockdowns, particularly for individuals self-regulated in an autonomous manner, presents profound implications that warrant attention. An essential contribution of this study is to highlight the possible role of positive affect in buffering anxiety levels associated with autonomous regulation. It underscores the pivotal role of exercise professionals in fostering positive emotions and creating environments that minimize the potential for inducing negative affect, given the observed relationship between affective states and anxiety. Moreover, this study sheds light on the evolving fitness landscape, with a growing trend toward online training. It underscores the need for fitness establishments, including gyms, to develop strategies ensuring that training environments, whether in-person or online, remain conducive to positive emotional experiences for practitioners. While online training has become a reality for many, the psychosocial consequences of these shifts, particularly concerning practitioners' well-being and anxiety levels, demand thorough examination in future studies. A comprehensive analysis, involving larger samples, has the potential to provide nuanced insights into the psychosocial implications of emergent fitness trends.

Furthermore, the study underscores the significance of contextual support in promoting autonomous motivation. Exercise professionals, guided by the principles of self-determination theory, should prioritize creating environments that facilitate autonomous motivation that contributes to positive affective experiences among practitioners. Additionally, the emphasis on creating environmentally friendly contexts within gym settings is highlighted as a crucial factor in sustaining the appeal and accessibility of in-person exercise. These considerations are pivotal for navigating the evolving fitness context and ensuring the continued promotion of psychosocial well-being in response to changing exercise modalities and contexts.

## Conclusion

The results of the present study revealed a positive association between autonomous motivation and positive affect, and a negative association between autonomous motivation and negative affect. Moreover positive, and negative affect are negatively and positively associated to anxiety, respectively.

This study has illuminated the intricate relationships among autonomous motivation, affective states, and anxiety within the unique context of the COVID-19 lockdown. The findings underscore the importance of considering psychosocial dimensions, particularly anxiety, in the context of individuals' confinement, especially those with a autonomous regulatory style in exercise. Positive affect emerges as a crucial buffer against anxiety, emphasizing the need for exercise professionals to curate environments that foster positive emotions and avoid those that may induce negativity.

## Data Availability

The information used in this research was acquired through a specialized license solely for the purpose of this study. The data that underpins the conclusions of this research is not accessible to the public but can be requested and obtained through reasonable inquiry, pending approval from the corresponding author.
