# Author Correction: Exploring the relationship across autonomous motivation, affects, and anxiety among gym practitioners during the second COVID-19 lockdown

**DOI:** 10.1038/s41598-024-60231-2

**Published:** 2024-04-23

**Authors:** Raúl Antunes, Filipe Rodrigues, Miguel Jacinto, Nuno Amaro, Rui Matos, Diogo Monteiro

**Affiliations:** 1 ESECS - Polytechnic University of Leiria, Leiria, Portugal; 2 Research Center in Sport Sciences, Health Sciences and Human Development (CIDESD), Vila Real, Portugal; 3 Life Quality Research Centre, Leiria, Portugal

Correction to: *Scientific Reports* 10.1038/s41598-024-57878-2, published online 27 March 2024

The original version of this Article omitted an affiliation for Filipe Rodrigues. The correct affiliations are listed below.

ESECS - Polytechnic University of Leiria, Leiria, Portugal.

Research Center in Sport Sciences, Health Sciences and Human Development (CIDESD), Vila Real, Portugal.

Life Quality Research Centre, Leiria, Portugal.

In addition, the Funding section was incomplete.

“This work was funded by National Funds by FCT—Foundation for Science and Technology under the following project UIDB/04045/2020 (https://doi.org/10.54499/UIDB/04045/2020).”

now reads:

“Work financed by national funds through FCT – Foundation for Science and Technology, I.P., under the project nº UID/CED/04748/2020 and UIDB/04045/2020 (https://doi.org/10.54499/UIDB/04045/2020).”

The original Article has been corrected.

